# A Manual of Operative Surgery

**Published:** 1886-03

**Authors:** 


A Manual of Operative Surgery. By Lewis A. Stinson, B. A., M. D., Surgeon to the Presbyterian and Bellevue Hospitals, Professor of Clinical Surgery in the Medical Faculty of the University of the City of New York, Corresponding member of the Societe de Chirurgie of Paris. Second Edition, with three hundred and fifty-two illustrations. Philadelphia: Lea Brothers & Co., 1885.

The opening chapter on anæsthesia does not include in the list of agents the bromide of ethyl, which has given satisfactory results with Chisolm and others, and the preliminary hypodermic use of morphine and atropia is overlooked, though generally recognized as modifying favorably the influence of chloroform and ether.

In the antiseptic treatment of surgical wounds, the use of solutions of corrosive sublimate to wash the wound is recommended, notwithstanding the serious effects which are known to have ensued from its absorption. The difficulties presented in ligation of the subclavian artery may be obviated in most cases requiring opiates by ligation of the artery below the clavicle by the French process, and this requires less discrimination in anatomical details.

In describing the circular operation for amputation there is no reference to rounding off the projections at each end of the linear union, which has been found a great improvement, not only for the appearance of the stump, but in obviating sloughing of those corners.

The suggestions of Rizzoli and Esmarch for division of the inferior maxillary, or removal of a wedge-shaped piece of the bone in ancylosis from adhesions left behind by intrabuccal ulceration, are endorsed when the cause itself cannot be removed. But this solution of the difficulty could only be warranted after a faithful effort to dissect out the cicatricial bone from its bony attachments when no osseous union exists between the condyle and temporal bone. No allusion is made to the relief of a bony union between the coronoid process and zygomatic arch, which the writer has separated with saw and chisel, thus securing complete mobility of the articulation.

There is no provision made in complete resection of a considerable segment of the tibia with its periosteum for the removal of a corresponding extent of the fibula, so as to approximate the osseous surface of the tibia. This, of course, involves shortening, but as the author remarks in regard to resection of the femur, "shortening is less of an infirmity than pseud-arthrosis."

In treating of trephining, the omission to notice the conical screw trephine, commended by Galt for security against injury to the brain, may be intentional, but is a defect in a practical point of view.

A successful case of gastrostomy by a North Carolina surgeon has escaped the attention of the author in the very small number noted, and the plan of inflating the stomach to enable the operator to find it when empty is calculated to obviate the difficulties encountered in gastrostomy by the author in locating it.

The cuts of a formidable apparatus with pins in pieces of cork within the lumen of the intestinal canal might have been substituted by a presentation of the Bishop suture for uniting the intestinal walls.

Unnecessary complications are made in the simple operation of circumcision in proposing to slit up the cutaneous prepuce above and below, or again to resort to the process of Delavan. This is an objection in a work on operative surgery intended to be a guide to others.

These points, with some others, have attracted our attention in examining this really useful manual, and are only exceptions to the general rule of excellence which characterizes it.

The drawings are for the most part admirably executed, and the descriptions plain and practical, while the printing and binding are all that could be desired, being in keeping with the recognized high standard of work by Lea Brothers & Co.

